# The impact of emojis on perceived responsiveness and relationship satisfaction in text messaging

**DOI:** 10.1371/journal.pone.0326189

**Published:** 2025-07-02

**Authors:** Eun Huh

**Affiliations:** Department of Communication Studies, The University of Texas at Austin, Austin, Texas, United States of America; Universitat Autònoma de Barcelona: Universitat Autonoma de Barcelona, SPAIN

## Abstract

Partner responsiveness is a key predictor of likability and overall relationship quality, yet its role in digital communication remains insufficiently studied. This study examined how the inclusion of emojis in text messages influences perceptions of responsiveness and, subsequently, likability, closeness, and relationship satisfaction. A sample of 260 participants viewed 15 text-based conversations in which they assumed that they received responses from their friends. Participants were randomly assigned to one of two conditions (e.g., text-only responses or responses that combined text and emojis) for each conversation. Perceptions of responsiveness, likability, closeness, and relationship satisfaction were assessed using pre-established Likert scales. The results showed that messages containing emojis were perceived as more responsive than text-only messages. Perceived responsiveness, in turn, significantly predicted higher ratings of closeness and relationship satisfaction. These findings demonstrate that the influence of partner responsiveness, enhanced through the use of emojis, extends beyond face-to-face interactions into digital interactions. They highlight the critical role of perceived responsiveness in digital contexts and suggest that emojis can enhance relationship satisfaction by acting as nonverbal cues that complement or substitute face-to-face communication.

## Introduction

Text messaging is the primary use of smartphones, with near-universal adoption across age groups. With 100% of individuals aged 18–29 using their smartphones for texting [1], text messaging has become ubiquitous. The use and variety of emojis have also surged, particularly among young adults. Emojis, as digital symbols conveying nonverbal cues, can be seen as a modern extension of long-standing practices enhancing digital interpersonal communication [2]. Emojis are used over 10 billion times daily worldwide [3,4], with 8 in 10 people incorporating emojis into their digital interactions [5]. Women and younger individuals tend to use emojis more frequently than older adults [6,7].

Defined as digital representations of emotions and ideas [8], emojis enrich text-based communication by adding emotional nuance and visual context. Face emojis, such as 
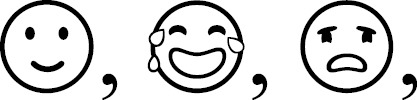
 are particularly popular for clarifying or enhancing the emotional tone of messages [9,10]. Viewed through the lens of metacommunication, emojis function similarly to earlier methods such as underlining, bolding or capitalization, which have historically been used to add emotional or interpretive cues to text [11]. Within the field of computer-mediated communication (CMC), the seminal work of Walther [12] proposed the Social Information Processing Theory, which suggests that our interactions and exchanges within online contexts can serve relatively equivalent functions to in-person ones by enabling individuals to obtain social information and support interpersonal processes. According to the Social Information Processing Theory, people use available behavioral cues to develop relationships and interpret others’ perspectives [12,13]. Building on this, Walther [12] introduced the Hyperpersonal Model, which suggests that online communication can surpass face-to-face (FtF) communication in relational depth due to the tendency to overcompensate for the lack of nonverbal cues through more selective self-presentation and carefully crafted messages. Emojis, as paralinguistic and nonverbal digital cues, enrich these “less rich” communicative exchanges by conveying emotional nuance, increasing expressiveness, and fostering relationship development [14]. While emoji meanings may vary across languages and cultures [15], research suggests that their interpretation can also exhibit cross-cultural consistency [15], making them valuable tool for cross-cultural communication [16]. To maintain relevance and inclusivity, the Unicode Consortium regularly updates emoji options based on user suggestions [17].

Emojis provide several advantages over text-based communication. As visual and non-linguistic cues, they enhance information processing [18], helping recipients interpret specific words more accurately or clarify ambiguous messages [19]. Additionally, emojis elicit neural responses similar to those in FtF interactions, making them effective in CMC [20,21]. Given that the human brain can recognize images in just 13 milliseconds, emojis are a quick yet effective communication tool [22]. They are also efficient, as they can be sent with a single touch, simplifying and speeding up communication [23]. As an evolution of longstanding practices in written communication, emojis are an easy and intuitive way to express emotions [24]. Although providing a quick and simple way to express emotions, emojis add emotional nuance and nonverbal cues to text-based interactions, which help clarify intentions, reduce misunderstandings, and foster stronger emotional connections [13,25].

However, the impact of emojis is not always positive. While they often enhance emotional clarity and message depth [20,26], emojis can also introduce ambiguity, especially in contexts with multiple possible interpretations, such as sarcasm [27]. In intimate communication, emojis may sometimes lead to confusion or misinterpretation [28]. Although emojis reflect a modern twist on established communication techniques, their potential to fundamentally alter interpersonal dynamics warrants further investigation. Although emojis are prevalent in texting and social media, their potential as a tool for fostering interpersonal connection remains underexplored [9].

**Fig 1:**
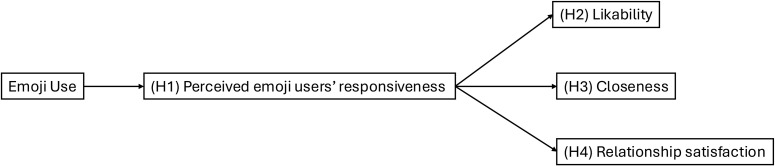
Overview of the Current Study.

This study investigates how emojis influence relationship perceptions in an era dominated by online interactions. In particular, the present study focuses on interactions with close friends and uses interaction scenarios to experimentally demonstrate that the use of emojis mediates improvements in responsiveness and enhances likability, closeness, and relationship satisfaction (Fig 1). Although the term *partner* is used throughout the paper, it refers specifically to a close friend with whom participants are exchanging text messages. Furthermore, the study also examines the distinct effects of face and non-face emojis, which differ in expressiveness [29], to assess whether certain types of emojis are more effective in facilitating positive interpersonal perceptions. Prior research suggests that face emojis – such as smiling faces or hearts – are more closely tied to emotional expression and can mimic facial expressions in FtF communication, thereby enhancing emotional clarity and social presence [13,27]. In contrast, non-face emojis – such as objects or symbols – can serve more diverse pragmatic purposes, such as indicating shared context. By examining this distinction, the study explores whether the expressive richness of face emojis results in stronger effects on relational perceptions compared to non-face emojis.

This study makes several contributions to the field of communication research. First, the study provides insights into how individuals navigate relational expectations from their friends’ emoji use. Individuals might seek signs of attentiveness and emotional engagement from their friends. By adding nuance and warmth to text-based interactions, emojis can signal effort and investment in conversations. Thus, understanding how emojis function in these interactions provides valuable insights into the social and emotional dynamics of online friendships. Second, the study deepens the understanding of how emojis function as digital expressions that can enhance emotional communication in text messaging. The study underscores the role of emojis as an important development in a continuum of practices that enrich written communication as emojis remain a widely used and efficient means of conveying emotional nuances in text-based communications. By distinguishing between face and non-face emojis, this study highlights the nuanced role of these visual cues in shaping perceptions of partner responsiveness and further relational outcomes. As simple pictorial and ready-made cues, emojis can contribute to and even enhance relational perceptions by efficiently conveying emotional nuances and nonverbal signals, fostering deeper emotional connections in digital interactions.

## Literature review

### Emojis in CMC

Positive and effective communication is essential for reducing relationship uncertainty and fostering confidence, closeness, and commitment between partners [30]. CMC, particularly through text messaging, plays a significant role in maintaining relationships [31]. Research shows that CMC can be as effective as FtF communication in reducing relationship uncertainty [32]. Additionally, emojis can enhance clarity in general online interactions [33] and romantic conversations [34]. CMC also facilitates the sharing of personal information, which helps partners feel more connected [35], thereby increasing intimacy [36] and improving overall relationship quality [37,38].

Historically, individuals have employed various strategies to compensate for the absence of nonverbal cues in written communication or in CMC, such as underlining, capitalization, exclamation marks, or even hand-drawn symbols like smiley faces. Emojis, particularly facial ones, may not constitute an entirely novel mode of expression but rather a 21st-century update to these traditional practices. This perspective underscores the continuity of human efforts to enhance textual communication with nonverbal elements and invites further exploration into how emojis extend or transform these established metacommunicative functions.

Emojis are often used by individuals to create a sense of similarity and synchronize the length, frequency, and timing of text exchanges [39]. In online dating contexts, mutual interest is frequently gauged by observing patterns of synchronized emoji use, which can encourage further interaction [40]. While emotionally expressive text responses typically convey greater interest and positivity, emojis can sometimes introduce ambiguity or even negativity in serious conversations, potentially signaling reduced interest [28]. As a result, the impact of emoji use in CMC on relationships is context-dependent and warrants further research to understand how specific uses of emojis can enhance or hinder relational outcomes.

Emojis do not always enhance digital communication. Their presence may lead to longer reading time [41] and may reduce message credibility and cognitive processing [42]. While positive emojis generally enhance perceptions of humor and positivity compared to a message with a negative emoji [43], they do not always increase perceived joy [44]. In business settings, emoji use can improve customer satisfaction but may also lower the perceived competence of the service employees using emojis, eventually impacting service satisfaction negatively [45,46]. In online consumer reviews, emojis may reduce perceived review credibility [25] and weaken the persuasiveness of negative reviews [47]. These findings highlight the nuanced effects of emojis across different communication contexts, underscoring the need for further research into their role in shaping digital interactions.

Although there has been extensive research on face emojis, non-face emojis remain relatively underexplored [48]. Face emojis primarily express emotions, while non-face emojis are often used to convey implicit textual meanings [49]. Moreover, the two types of emojis differ significantly in how they are interpreted and understood [50]. Therefore, the present study examines both types of emojis.

### Perceived responsiveness of emoji users

Perceived partner responsiveness enhances both relationship satisfaction and communication quality across various communication media [51]. It influences not only an individual’s self-perception but also evaluations of the conversational partner and the topic being discussed. Positive regard from others, such as responsiveness, bolsters self-evaluations, and momentary self-esteem, particularly for individuals with high social anxiety [52,53]. Furthermore, reciprocal responsiveness increases positive mood and reduces negative mood, while a lack of reciprocation can intensify negative emotions [54].

Perceived partner responsiveness also affects evaluations of conversation topics and overall communication outcomes. For instance, positive feedback from strangers during initial interactions leads to more favorable immediate and long-term memories of the interaction [55]. In romantic relationships, responsiveness plays a critical role in shaping partner perceptions and emotional well-being. Positive emotion words and Language Style Matching (LSM) help individuals navigate emotionally significant events, enhancing their well-being [56]. Additionally, responsiveness enhances post-interaction perceptions in romantic relationships and is associated with better sleep quality, as it alleviates anxiety [57,58]. For individuals with low self-esteem, responsiveness strengthens commitment by influencing relational factors such as investment and expressivity [59,60]. Longitudinal studies further reveal that feeling understood, cared for, and appreciated by a romantic partner supports better stress regulation, as evidenced by improved cortisol levels over a decade [61].

In the context of online communication, perceived partner responsiveness remains highly influential. On online forums, for instance, the frequency of responses to users’ posts predicts public interest and engagement in discussions [62]. Similarly, on social media, likes and comments evoke strong emotional reactions. Individuals feel happier and more satisfied when their posts receive more engagement than expected, perceiving this feedback as positive audience validation [2]. Conversely, insufficient peer engagement, such as a lack of likes or positive comments on selfies, can cause stress among teenage girls. This often leads them to adopt emotion- and problem-focused coping strategies [45,63].

### Emojis and perceived responsiveness

Responsiveness, whether directed at positive or negative information, involves providing active attention to the self-concept of the individual sharing the information [64]. Effective responsiveness is characterized by a partner’s perceived understanding, validation, and care [65]. In CMC, where nonverbal cues like voice tone, facial expressions, and gestures are absent, paralinguistic elements like emojis play a crucial role in conveying responsiveness [57]. Emojis enhance the recipient’s understanding by intensifying, negating, or clarifying the emotional intent of a written message [43,66,67].

Compared to simple emoticons (e.g.,:) ), emojis provide more complex and nuanced expressions, serving multiple functions in CMC. They assist in modifying the tone of the message, managing the flow of conversations, and reducing interpersonal distance [68, 69, 70]. By making exchanges feel more personal and less formal, emojis help bridge social gaps and demonstrate thoughtfulness toward the recipient [71]. Beyond emotional expression, emojis also serve decorative purposes, enhance a message’s visual appeal, and evoke stronger emotional responses [68]. They are particularly useful for signaling message receipt when there is little else to add in response [69]. Given the diverse functions and benefits of emojis in digital communication, the author hypothesizes that:

**Hypothesis 1 (H1)** Emoji use will enhance one’s perceptions of the emoji user’s responsiveness.

### Likability

Likability, a key component of social exchange, is defined as the ability to create positive emotional experiences and put others at ease [72]. Individuals who engage in intimate self-disclosures are generally perceived as more likable than those who disclose less [73]. Similarly, responsiveness is a critical factor influencing likability; responsive individuals are consistently rated more favorably than unresponsive ones [74]. Both men and women value responsiveness as a vital trait in potential romantic partners [52,75]

Emojis, as paralinguistic elements, can significantly influence how messages and their senders are perceived. For instance, responsiveness shapes perceptions of both human and non-human communicators: animated figures displaying responsive behaviors, such as head nodding, are rated as more likable and approachable [76]. Emojis similarly enhance communication by making it more positive and efficient, often increasing the sender’s likability [33,77]. For example, a male manager who included an emoji in a work email was perceived as warmer than when no emoji was used [78]. Likewise, instructors incorporating emojis in their emails tend to be viewed more favorably by students [79], although they may be viewed as less competent when smiling emoticons are used [80]. Non-face emojis, in particular, can improve message clarity and enhance positivity [33,81]. Perceptions of likability influenced by emoji use are also shaped by factors such as gender and the type of emoji used. For example, affectionate emojis from women are seen as more appropriate and likable, while friendly emojis from men are viewed as equally appropriate and contribute to greater likability [82]. Based on these findings and the established link between perceived partner responsiveness and likability, the author proposes the following hypothesis:

**Hypothesis 2 (H2)** An individual’s perceptions of an emoji user’s responsiveness will be positively associated with the individual’s perceptions of the emoji user’s likability.

### Closeness

Perceived partner responsiveness enhances an individual’s sense of intimacy and strengthens feelings of closeness within relationships [83], in which closeness is often viewed as the overlap of individuals within that relationship [84]. This responsiveness can stem from expressing gratitude [85], sharing positive events [86], and even disclosing negative experiences [57]. When individuals perceive their partners as responsive during communication, this fosters greater intimacy and closeness, which further reinforces partner responsiveness [87]. Additionally, perceived partner responsiveness facilitates feelings of closeness and promotes open communication, even during stressful times [88,89].

Nonverbal cues, such as gestures and facial expressions, are essential for effectively conveying responsiveness in FtF interactions [57]. In CMC, emojis serve as an effective substitute for nonverbal cues, allowing users to express emotions and signal responsiveness [9]. For instance, using emojis to disclose positive events has been shown to enhance perceived responsiveness [9]. Based on these insights, the author hypothesizes the following:

**Hypothesis 3 (H3)** An individual’s perceptions of an emoji user’s responsiveness will be positively associated with the individual’s perceptions of closeness with an emoji user.

### Relationship satisfaction

Perceived partner responsiveness is a key determinant of relationship satisfaction, as it fosters intimacy [83] and enhances marital satisfaction [26]. Beyond its role in general satisfaction, responsiveness has been linked to improved mental and physical outcomes. For instance, it predicts lower levels of depressive symptoms over time, particularly in individuals with a high need for emotional expression [90]. In a study on knee osteoarthritis patients, those with empathically responsive spouses experienced better physical functioning over time compared to those with less responsive partners [91]. Similarly, in parent-child relationships, parental responsiveness mediates the connection between a child’s self-disclosure and the quality of the parent-child bond [92].

Perceived partner responsiveness is closely tied to perceptions of relationship satisfaction. Positive, responsive behaviors contribute to greater marital satisfaction, which is positively associated with emotional support and negatively associated with conflict [93]. Interventions that promote enthusiastic responses, rather than simply engaging in joint activities, significantly enhance partner satisfaction [42]. However, differences exist across age groups, as older married couples tend to engage in fewer responsive listening behaviors compared to middle-aged couples [94]. Notably, even when actual responsiveness is low, the mere perception of responsiveness can elevate relationship satisfaction [95]. Higher relationship satisfaction is also linked to overall happiness and life satisfaction [96,97]. Conversely, negative affect reciprocity – where negative behaviors are reciprocated – predicts relationship dissatisfaction and may lead to relationship dissolution [98].

In digital communication, perceived responsiveness remains a crucial factor in relationship satisfaction. Positive text messaging between partners is associated with higher satisfaction levels [31]. Customized, affectionate messages containing positive emotional language predict long-term relationship satisfaction [31,99]. Emojis, in particular, enhance the expression of affection and maintain emotional connection in text-based communication, thereby strengthening relational bonds [14]. Additionally, synchronizing punctuation and emoji use improves relational communication [9]. Likewise, reciprocating emoji use (vs. no emoji) results in higher perceptions of warmth, playfulness, and communication positivity [100]. Furthermore, regardless of the valence of the message (i.e., positive, neutral, or negative), individuals who use positive emojis (vs. negative; vs. neutral; vs. no emoji) were always perceived as warmer [79]. Based on these findings, the author hypothesizes that:

**Hypothesis 4 (H4)** An individual’s perceptions of an emoji user’s responsiveness will be positively associated with the individual’s perceptions of relationship satisfaction with the emoji user.

## Methods

The present study was approved by the Institutional Review Board at the University of Texas at Austin. The study employed a between-subjects online experimental design. 15 stimulus materials were created, each consisting of four text exchanges that varied only in the presence or absence of emojis while maintaining identical textual content. Participants were randomly assigned to one of the two experimental conditions (i.e., a text-only condition and a text-and-emoji condition) for each message. After reviewing each conversation, participants rated the responder on perceived responsiveness. Additionally, they evaluated the responder’s interpersonal qualities, including likability, closeness, and relationship satisfaction.

### Participants

Participants were recruited through Amazon Mechanical Turk (MTurk), an online platform where individuals complete tasks in exchange for monetary compensation. Initially, 300 participants were recruited, each receiving payment upon survey completion. Responses from participants who failed any attention check questions or who did not complete the survey were excluded, resulting in a final sample size of 260. All participants were proficient in English and located in the United States.

For hypothesis 1, a power analysis specifying a medium effect size (ƒ = .25) indicated that for a two-sample t-test, a total sample size of 128 (64 per group) would be needed. For hypotheses 2, 3, and 4, the author had no prior findings to rely on for estimating power, therefore assuming small to medium effect sizes. Since these hypotheses involved mixed-effects modelling, a simulation-based power analysis was conducted. Results indicated that, even with an assumed small-to-medium effect size (*β* = 0.18), the model demonstrated high statistical power (100%, 95% CI [98.59%, 100%]) to detect the effect of the predictor, suggesting sufficient sensitivity to test the proposed hypotheses.

The majority of participants identified as White/Caucasian (*n* = 243, 93.5%), with smaller proportions identifying as Black/African American (*n* = 8, 3.1%), Asian/Pacific Islander (*n* = 5, 1.9%), and Native American/American Indian (*n* = 3, 1.2%). Most participants (62.7%, *n* = 163) identified as male, and 62.3% (*n* = 162) reported holding a bachelor’s degree. The average participant age was 37.32 years (*SD *= 8.38), ranging from 23 to 67 years.

### Experimental design and procedure

MTurk workers aged 18 or older who reside in the United States were invited to participate via a brief description posted on the MTurk platform from August 28th, 2024 to August 31st, 2024. Interested participants were directed to a Qualtrics survey, where they read and electronically provided written informed consent. The recruitment process took place exclusively during this period, and all participants provided written consent before starting the study.

Participants were asked to read 15 text message exchanges between close friends in an informal context, as emojis are typically used in such settings [101]. They were instructed to imagine themselves as the sender of each message (displayed in blue speech bubbles) while focusing on their partner’s replies (displayed in grey speech bubbles) to evaluate aspects of the relationship. An example conversation, featuring a “Hello” message exchange, was presented to familiarize participants with the format, where sent messages were labeled as “sent” and received messages as “received.”

Each participant was randomly assigned to one of two versions of each conversation: (a) text-only (no emoji) format (replies consisted solely of text), or (b) emoji format (replies included both text and emojis). Thus, participants viewed each conversation in only one format. After each conversation, participants rated their perceptions of the following relationship dimensions after each conversation: (a) partner responsiveness, (b) closeness, (c) likability, and (d) relationship satisfaction.

After rating all 15 conversations, participants completed demographic questions on gender, ethnicity, age, and education. Additionally, they rated their frequency of emoji use in daily CMC and texting on a 7-point Likert scale (1 = *Never*, 7 = *Always*). Upon completion, participants received a unique four-digit code to confirm participation on the MTurk site and receive payment.

### Stimulus material

For the present study, 15 text conversations were created to explore the influence of emojis. These conversations included: (a) face emojis (e.g.,
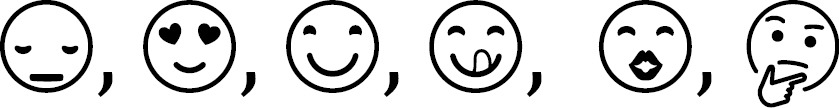
) and non-face emojis (e.g., 
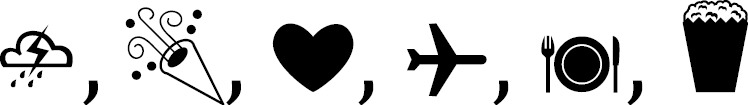
). Facial emojis express various positive and negative emotions, making them central to online emotion expression. Non-face emojis were also included because some have links to emotional affect [102], yet they are less frequently examined in research compared to facial emojis [103,104]. Thus, investigating both types allows a more comprehensive understanding of how different emoji forms contribute to digital communication. Out of 15 text conversations, eight (53.3%) included face emojis, while seven (46.7%) featured non-face emojis.

Importantly, all emoji-text combinations were intentionally designed to be congruent in emotional valence. That is, positive emojis were paired with positively valenced text, negative emojis with negatively valenced text, and neutral emojis with neutral text. This approach ensured consistency in emotional tone and reduced the possibility of misinterpretation, sarcasm, or incongruence between textual and visual cues. To account for the contextual factors influencing emoji interpretation [69], participants were instructed to imagine exchanging messages with their close friends. All stimulus materials were presented in English.

Each conversation began with either a statement (e.g., “It’s been a long week. I’m tired.”; *n* = 7) or a request (e.g., “Hey, I was thinking we could go out for dinner tonight. How about the Italian place we love?”; *n* = 8). Responses were presented in two formats: (a) text-only format: contained written content only (e.g., “You got promoted? Congrats! We should celebrate tonight!”) and (b) emoji format: included both text and emojis (e.g., “Congrats 
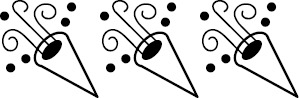
 We should celebrate tonight!”).

**Fig 2:**
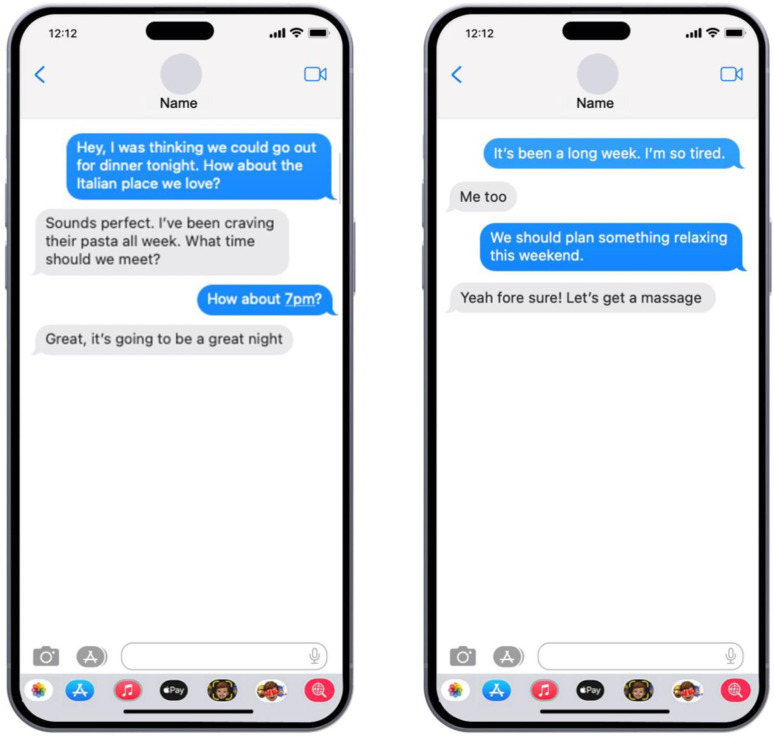
Examples of Stimuli. A text-only condition (Statement-opener and request-opener).

**Fig 3:**
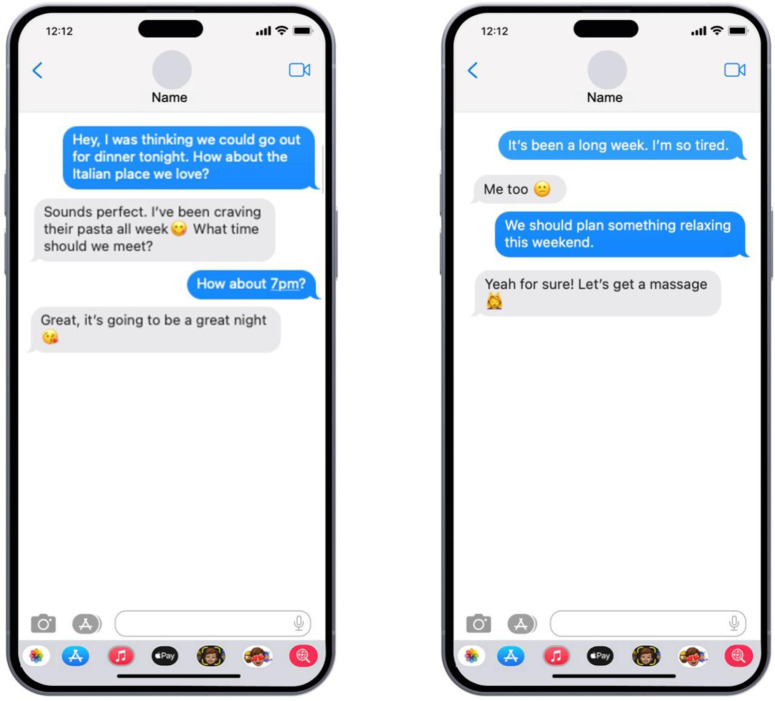
Examples of Stimuli. An emoji and text condition (Statement-opener and request-opener).

The conversations were displayed in random order. Messages were presented as standard mobile screen images, replicating a typical messaging app layout with sent messages in blue bubbles and received messages in grey. To control for confounding variables, sent messages contained no emojis, while received messages included emojis. See Fig 2 for the example of a text-only condition and Fig 3 for the example of an emoji and text condition.

The selection of conversation scenarios and emoji options used in the experiment was based on a pilot study with a group of undergraduate students (N = 12). The initial pool of 25 conversation scenarios was developed by the author based on common patterns of everyday interpersonal communication. Scenarios were designed to vary in emotional expressiveness and familiarity, drawing from typical themes in informal conversations (e.g., making plans, offering support, sharing news). In the pilot study, undergraduate participants rated each scenario on a 7-point Likert scale in terms of how realistic, familiar, and emotionally expressive each message felt. Based on these ratings, the 15 highest-scoring scenarios were selected for inclusion in the main study. This process ensured that the final stimulus materials reflected communication patterns that participants would likely encounter in everyday life, enhancing ecological validity. Additionally, the emojis used in the final stimuli were those most frequently endorsed by the pilot participants as appropriate and emotionally resonant for each context.

### Measures

The scales used in the present study were adapted from prior research, which employed different response formats (i.e., 5-point and 7-point scales).

#### Perceived partner responsiveness.

Participants rated the statement, “My partner is being responsive to me” on a 5-point Likert scale ranging from 1 (*not responsive at all*) to 5 (*very responsive*) (α = .85). While prior research has typically measured perceived partner responsiveness using multi-item scales [64], single-item measures have been shown to perform adequately when the construct is unambiguous and clearly understood by participants [105]. The present study focuses on immediate impressions of responsiveness within short, simulated message exchanges, making a single-item format appropriate and efficient for capturing participants’ perceptions.

#### Likability.

Partner likability was measured using four items on a 7-point bipolar scale, adapted from Jones et al. [106], and previously employed in studies examining email recipients’ impressions of senders [107]. Items included: “not friendly at all (1) - very friendly (7),” “not understanding at all (1) - very understanding (7),” “not likable at all (1) - very likable (7),” and “not respectable at all (1) - very respectable (7)” (α = .92).

#### Closeness.

Closeness was assessed using the Inclusion of the Other in the Self (IOS) scale [84], a 7-point pictorial scale illustrating two overlapping circles labeled “You” and “Your Friend.” Participants selected the degree of overlap to indicate perceived relationship closeness (1 = *no overlap*, 7 = *most overlap*). The measure showed high reliability (α = .93).

#### Relationship satisfaction.

Relationship satisfaction was evaluated using an adapted version of the Relationship Assessment Scale (RAS) [108]. Participants responded to seven items on a 5-point scale with varying anchors. Example items included: “How often do you think this person will meet your needs? (1 = *poorly*, 5 = *extremely well*)” and “In general, how satisfied will you be with your relationship? (1 = *unsatisfied*, 5 = *extremely satisfied*).” This scale demonstrated acceptable reliability (α = .73).

## Results

Table 1 includes all means and standard deviations for perceived responsiveness, likability, closeness, and relationship satisfaction across conditions. Table 2–3, and Table 4 present the correlation matrix for the key study variables. Table 2 shows the overall correlation matrix (combining face and non-face emoji conditions), Table 3 displays the matrix for the face emoji condition, and Table 4 presents the matrix for the non-face emoji condition.

**Table 1:** Means and Standard Deviations Across Experimental Conditions.

Variables	No emoji	Emoji		
		Overall	Facial emojis	Non-face emojis
Responsiveness	3.57 (.93)	4.43 (.71)	4.42 (.72)	4.45 (.69)
Likability	5.55 (.94)	5.65 (.94)	5.60 (.98)	5.71 (.89)
Closeness	4.83 (1.35)	5.06 (1.31)	5.06 (1.33)	5.06 (1.28)
Relationship satisfaction	3.93 (.51)	3.96 (.52)	3.96 (.52)	3.95 (.52)

*Note.* The mean scores and standard deviations of the variables are presented.

**Table 2:** Correlation Matrix for Overall Emoji Condition.

	1	2	3	4
1 Responsiveness	–			
2 Likability	.24	–		
3 Closeness	.26	.23	–	
4 Relationship satisfaction	.27	.66	.18	–

**Table 3:** Correlation Matrix for Face Emoji Condition.

	1	2	3	4
1 Responsiveness	–			
2 Likability	.22	–		
3 Closeness	.29	.22	–	
4 Relationship satisfaction	.23	.64	.15	–

**Table 4:** Correlation Matrix for Non-face Emoji Condition.

	1	2	3	4
1 Responsiveness	–			
2 Likability	.25	–		
3 Closeness	.30	.24	–	
4 Relationship satisfaction	.27	.66	.19	–

### Attention checks

Six attention-check questions were embedded throughout the survey to ensure participant attentiveness. These included three instructed response items (e.g., “In order to prove that you are reading questions attentively, please select ‘2’”) and three simple math questions (e.g., “What is 1 + 1? This is an attention check”). The attention-check items were based on established practices [109–112].

Of the 300 initial participants, 260 (86.7%) passed all six attention checks and were included in the final analysis. The remaining 40 participants (13.3%) who failed one or more attention checks were excluded.

### Perceived partner responsiveness

Hypothesis 1 (H1) predicted that emoji use significantly enhances one’s perceptions of an emoji user’s responsiveness. A two-sample t-test supported H1, revealing a statistically significant difference in perceived responsiveness between emoji users and non-emoji users, *t*(258) = 8.90, *p* < .001, Cohen’s *d* = 1.05. Participants rated emoji users more responsive (*M* = 4.43, *SD* = .71) than non-emoji users (*M* = 3.57, *SD* = .93).

To further investigate whether the type of emoji (face vs. non-face) influenced perceptions of responsiveness, another two-sample t-test was conducted. The results indicated no significant difference in perceived responsiveness between face (*M* = 4.42, *SD* = .72) and non-face emojis (*M* = 4.45, *SD* = .69), *t*(258) =.86 *p* = .39.

Overall, the findings demonstrate that using emojis significantly enhances perceptions of responsiveness compared to not using emojis. However, the specific type of emoji – whether a face or a non-face symbol – does not influence perceived responsiveness. This suggests that the presence of emojis alone, regardless of their specific type, is sufficient to positively shape perceptions of responsiveness.

### Likability

A series of linear mixed-effects models were conducted to examine whether perceived partner responsiveness predicted likability, closeness, and relationship satisfaction, while accounting for random intercepts by emoji presence.

Hypothesis 2 (H2) suggested that individuals’ perceptions of an emoji user’s responsiveness are positively associated with individuals’ perceptions of the emoji user’s likability. The model revealed that perceived responsiveness did not significantly predict perceived likability, *b *= 0.18, *SE *= 0.04, *t*(258) = 4.28, *p* = .10. Thus, H2 was rejected.

Pearson correlations were conducted separately for the face emoji and non-face emoji groups. For the face emoji group, a weak positive correlation was found between perceived responsiveness and likability, *r* = .23, *t*(258) = 3.72, *p* < .001, indicating a slight increase in likability with increased responsiveness. For the non-face emoji group, a very weak positive correlation was observed, *r* = .16, *t*(258) = 3.86, *p* < .001. A *t*-test comparing the mean differences between the face and non-face emoji groups revealed no significant differences, *t*(258) = 1.70, *p* = .09.

The findings indicate that perceived responsiveness does not significantly predict perceived likability. While the correlation was slightly stronger for face emojis compared to non-face emojis, the difference was not statistically significant.

### Closeness

Hypothesis 3 (H3) proposed that individuals’ perceptions of an emoji user’s responsiveness are positively associated with feelings of closeness toward the emoji user. The model revealed that perceived responsiveness significantly predicted perceived closeness, *b *= 0.26, *SE *= 0.05, *t*(258) = 5.11, *p* < .001. Thus, H3 was supported.

Pearson correlations were conducted separately for the face emoji and non-face emoji groups. For face emoji group, a statistically significant positive correlation was found between perceived responsiveness and closeness, *r* = .27, *t*(258) = 4.51, p < .001, and for non-face emoji group, a s*t*atistically significant, albeit weaker, positive correlation was observed, *r* = .24, *t*(258) = 5.58, *p* < .001. A follow-up t-*t*est comparing the face and non-face emoji groups revealed no significant difference in perceived closeness between the two groups, *t*(258) =.70, *p* = .49.

The findings indicate that perceived responsiveness significantly predicts perceived closeness, with higher responsiveness associated with greater closeness. Both the face and non-face emojis demonstrated positive correlations between responsiveness and closeness, with a slightly stronger correlation for face emojis. However, this difference was not statistically significant, suggesting that the presence of emojis, regardless of type, plays a similar role in enhancing feelings of closeness.

### Relationship satisfaction

Hypothesis 4 (H4) predicted that one’s perceptions of an emoji user’s responsiveness is positively associated with one’s perception of relationship satisfaction with the emoji user. The model revealed that perceived responsiveness significantly predicted perceived satisfaction, *b *= 0.15, *SE *= 0.03, *t*(258) = 5.05, *p* < .001. Thus, H4 was supported.

Pearson correlations were conducted separately for the face emoji and non-face emoji groups. For the face emoji group, a statistically significant positive correlation was observed between perceived responsiveness and relationship satisfaction, *r* = .24, *t*(258) = 4.07, *p* < .001, and for *t*he non-face emoji group, a statistically significant positive correlation was found, *r* = .18, *t*(258) = 4.16, *p* < .001. A follow-up t-*t*est comparing the face and non-face groups revealed no significant difference in relationship satisfaction between the two groups, *t*(258) =.01, *p* = .99.

The findings indicate that perceptions of an emoji user’s responsiveness significantly predict relationship satisfaction, with higher responsiveness linked to greater satisfaction. Both the face and non-face emojis showed positive correlations between responsiveness and relationship satisfaction. While the correlation was slightly stronger for face emojis, the difference in relationship satisfaction between the two emoji types was not statistically significant.

### Mediation analysis

Three mediation analyses were conducted using Model 6 of the PROCESS macro to examine the indirect effects of emoji use on perceived likability, closeness, and relationship satisfaction, with perceived partner responsiveness as the mediator.

The first analysis examined the mediating role of perceived partner responsiveness in the relationship between emoji use and perceived likability. Nonparametric bootstrapping revealed a statistically significant indirect effect, *b* = .51, 95% C.I. [.46,.57], *p* < .001, indicating that perceived responsiveness mediated the relationship between emoji use and likability. The direct effect of emoji use on likability was not significant, *b* = .04, 95% C.I. [−.09,.02], *p* = .20, but the total effect was significant, *b* = .47, 95% C.I. [.42,.52], *p* < .001. The proportion of the total effect mediated by perceived responsiveness was 1.07, 95% C.I. [.96, 1.20], suggesting that the indirect effect accounted for most of the relationship. These results indicate that while emoji use alone did not directly influence likability, it enhanced the perceived responsiveness, which in turn increased likability ratings.

The second analysis investigated the mediating role of perceived partner responsiveness in the relationship between emoji use and perceived closeness. Nonparametric bootstrapping revealed a statistically significant indirect effect*, b* = .52, 95% C.I. [.44,.61], *p* < .001, showing that perceived responsiveness mediates the relationship between emoji use and closeness. The direct effect of emoji use on closeness was not significant, *b* = .01, 95% C.I. [−.09,.10], *p* = .93, but the total effect was significant, *b* = .53, 95% C.I. [.46,.59], *p* < .001. The proportion of the total effect mediated by perceived responsiveness was 1.00, 95% C.I. [.83, 1.18], indicating that the indirect effect accounted for most of the relationship. These findings suggest that emojis increase perceived responsiveness, which in turn enhances feelings of closeness.

The third analysis explored the mediating role of perceived partner responsiveness in the relationship between emoji use and perceived relationship satisfaction. Nonparametric bootstrapping revealed a significant indirect effect, *b* = .48, 95% C.I. [.44,.52], *p* < .001, demonstrating the mediating role of perceived responsiveness. The direct effect of emoji use on relationship satisfaction was also significant, *b* = .05, 95% C.I. [.03,.08], *p* < .001, and the total effect was significant, *b* = .43, 95% C.I. [.39,.47], *p* < .001. The proportion of the total effect mediated by perceived responsiveness was 1.12, 95% C.I. [1.06, 1.20], indicating that the indirect effect accounted for more than the total relationship between emoji use and relationship satisfaction. This suggests that emojis in text messages enhance perceived responsiveness, ultimately leading to higher relationship satisfaction.

The results from all three mediation analyses demonstrate that perceived partner responsiveness plays a crucial mediating role in the relationships between emoji use and perceived likability, closeness, and relationship satisfaction. While emoji use alone did not directly affect likability or closeness, its influence was fully mediated through perceptions of responsiveness. For relationship satisfaction, both direct and indirect effects were significant, highlighting the importance of responsiveness in shaping relationship outcomes.

**Fig 4:**
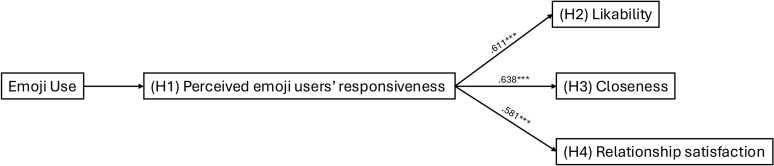
The mediation effect of perceived partner responsiveness on likability (H2), closeness (H3), and relationship satisfaction (H4). *Note*. ***p < .001.

Fig 4 visually illustrates the mediation effect for likability (H2), closeness (H3), and relationship satisfaction (H4).

### Moderation analysis

#### Frequency of emoji use.

Three moderation analyses were conducted to examine whether the relationship between emoji use and the participants’ perceptions of the three outcome variables was moderated by the participants’ self-reported frequency of emoji use.

For participants’ perception of emoji user’s likability, the overall model was significant, *F*(3, 256) = 104.40, *p* < .001, *R*^*2*^ = .55. The main effect of emoji use was significant, *b* = .42, *SE* = .17, *t*(258) = 2.43, *p* < .05], indica*t*ing that emoji use positively influenced perceptions of likability. However, the main effect of the participants’ self-reported frequency of emoji use was not significant, *b* = −.01, *SE* = .02, *t(258*) = −.57, *p* = .57. The interaction effect between emoji use and self-reported frequency of emoji use was also not significant, *b* = .01, *SE* = .03, *t(258*) =.27, *p* = .79, suggesting that self-reported emoji use frequency did not moderate the relationship between emoji use and likability.

A second moderation analysis examined the moderating effect of participants’ self-reported frequency of emoji use on the relationship between emoji use and participants’ perception of closeness with emoji users. The overall model was significant, *F*(3, 256) = 82.68, *p* < .001, *R*^*2*^ = .49. Neither the main effect of emoji use, *b* = .24, *SE* = .22, *t(258*) = 1.09, *p* = .28, nor the main effect of self-reported frequency of emoji use, *b* = −.02, *SE* = .03, *t(258*) = −.74, *p* = .46, was significant. The interaction effect between emoji use and self-reported frequency of emoji was not significant either, *b* = .05, *SE* = .04, *t(258*) = 1.33, *p* = .19. These findings suggest that participants’ self-reported frequency of emoji use did not moderate the relationship between emoji use and closeness.

A third moderation analysis was conducted to investigate whether the relationship between emoji use and participants’ perception of relationship satisfaction was moderated by participants’ self-reported frequency of emoji use. The overall model was significant, *F*(3, 256) = 127.90, *p* < .001, *R*^*2*^ = .60. The main effect of emoji use was significant, *b* = .39, *SE* = .14, *t(258*) = 2.71, *p* < .01, indicating a positive relationship between emoji use and relationship satisfaction. However, the main effect of self-reported frequency of emoji use was not significant, *b* = −.002, *SE* = .02, *t(258*) = −.14, *p* = .89. The interaction effect between emoji use and self-reported frequency of emoji use was not significant, *b* = .01, *SE* = .02, *t(258*) =.28, *p* = .78.The results across all three moderation analyses indicate that participants’ self-reported frequency of emoji use did not moderate the relationship between emoji use and perceived likability, closeness, or relationship satisfaction. While emoji use itself has significant effects on likability and relationship satisfaction, the frequency with which participants reported using emojis in their daily communication did not influence these relationships.

#### Age.

Another set of moderation analyses was conducted to examine whether the relationship between emoji use and the participants’ perceptions of the three outcome variables was moderated by the participants’ age.

The overall model was significant, *F*(3, 256) = 104.40, *p* < .001, *R*^*2*^ = .55. The main effect of emoji use was significant, *b* = .54, *SE* = .12, *t(258*) = 4.39, *p* < .001, indicating that emoji use positively influenced perceptions of likability. However, the main effect of the participants’ age was not significant, *b* = .00, *SE* = .00, *t(258*) =.52, *p* = .60. The interaction effect between emoji use and participants’ age was also not significant, *b* = .00, *SE* = .00, *t(258*) = −.54, *p* = .59, suggesting that participants’ did not moderate the relationship between emoji use and likability.

A second moderation analysis was conducted to examine whether age moderates the relationship between emoji use and participants’ perception of closeness with emoji users. The overall model was significant, *F*(3, 256) = 82.41, *p* < .001, *R*^*2*^ = .49. Results revealed a significant main effect of emoji use, *b* = .69, *SE* = .15, *t(258*) = 4.52, *p* < .001, indicating that individuals who were in the emoji condition reported greater closeness with emoji users than those in the no-emoji condition. However, participants’ age was not a significant predictor, *b* = .00, *SE* = .00, *t(258*) =.46, *p* = .65. The interaction effect between emoji use and age was not significant, *b* = .00, *SE* = .00, *t(258*) = −1.12, *p* = .26. These findings suggest that participants’ age did not moderate the relationship between emoji use and closeness.

A third moderation analysis was conducted to test whether age moderates the relationship between emoji use and participants’ perceptions of relationship satisfaction with the emoji user. The model was significant, *F*(3, 256) = 127.90, *p* < .001, *R*^*2*^ = .60. The main effect of emoji use was significant, *b* = .47, *SE* = .10, *t(258*) = 4.67, *p* < .001, indicating a positive relationship between emoji use and relationship satisfaction. However, the main effect of participants’ age was not significant, *b* = .00, *SE* = .00, *t(258*) = −.40, *p* = .80. The interaction effect between emoji use and participants’ age was not significant, *b* = .00, *SE* = .00, *t(258*) = −.40, *p* = .69.

The results across all three moderation analyses indicate that participants’ self-reported frequency of emoji use did not moderate the relationship between emoji use and perceived likability, closeness, or relationship satisfaction. While emoji use itself has significant effects on likability and relationship satisfaction, the frequency with which participants reported using emojis in their daily communication did not influence these relationships.

#### Gender.

Additional moderation analyses were conducted to examine whether the relationship between emoji use and the participant’s perceptions of the three outcome variables was moderated by the participants’ gender.

The overall model was significant, *F*(3, 256) = 107.30, *p* < .001, *R²* = .56. The main effect of emoji use was significant, *b* = 0.47, *SE* = 0.08, *t(258*) = 5.88, *p* < .001, indicating that emoji use positively influenced perceptions of likability. However, the main effect of the participants’ gender was not significant, *b* = 0.05, *SE* = 0.04, *t* (256) = 1.42, *p* = .16. The interac*t*ion effect between emoji use and participants’ gender was also not significant, *b* = 0.00, *SE* = 0.05, *t* (256) = 0.05, *p* = .96, suggesting *t*hat participants’ gender did not moderate the relationship between emoji use and likability.

A second moderation analysis was conducted to examine whether gender moderates the relationship between emoji use and participants’ perception of closeness with emoji users. The overall model was significant, *F*(3, 256) = 81.53, *p* < .001, *R²* = .49. Results revealed a significant main effect of emoji use, *b* = 0.51, *SE* = 0.10, *t(258*) = 5.06, *p* < .001, indicating that individuals in the emoji condition reported greater closeness with emoji users than those in the no-emoji condition. However, participants’ gender was not a significant predictor, *b* = 0.01, *SE* = 0.05, *t(258*) = 0.11, *p* = .91. The interaction effect between emoji use and gender was not significant, *b* = 0.01, *SE* = 0.07, *t(258*) = 0.14, *p* = .89. These findings suggest that participants’ gender did not moderate the relationship between emoji use and closeness.

A third moderation analysis was conducted to test whether gender moderates the relationship between emoji use and participants’ perceptions of relationship satisfaction with an emoji user. The model was significant, *F*(3, 256) = 129.60, *p* < .001, *R²* = .60. The main effect of emoji use was significant, *b* = 0.45, *SE* = 0.07, *t(258*) = 6.77, *p* < .001, indicating a positive relationship between emoji use and relationship satisfaction. However, the main effect of participants’ gender was not significant, *b* = 0.04, *SE* = 0.03, *t(258*) = 1.21, *p* = .23. The interaction effect between emoji use and participants’ gender was not significant, *b* = −0.01, *SE* = 0.05, *t(258*) = −0.24, *p* = .81.

The results across all three moderation analyses indicate that participants’ gender did not moderate the relationship between emoji use and perceived likability, closeness, or relationship satisfaction. While emoji use itself has significant effects on these relationship perceptions, gender did not influence these associations.

## Discussion

Interpersonal interactions encompass both face-to-face communication and digital exchanges. While relationship satisfaction can be enhanced through both forms, prior research has predominantly focused on relationship satisfaction within FtF contexts [83,26]. Furthermore, although the influence of nonverbal cues in digital interactions on relationship satisfaction has been examined, the effect of emojis on relationship satisfaction remains underexplored. Thus, this study seeks to fill this gap by examining how partner responsiveness operates within the context of digital communication.

The present study experimentally investigates how emoji use in text messages influences perceived partner responsiveness and, in turn, impacts perceptions of partner likability, closeness, and overall relationship satisfaction. Findings reveal that partners who used emojis were rated as more responsive than those who communicated through text alone. This suggests that emojis add emotional depth to messages and enhance partner engagement. Moreover, perceived partner responsiveness was positively associated with (a) closeness and (b) relationship satisfaction.

Importantly, the study found no significant differences in effects between face and non-face emojis. This finding suggests that the type of emoji may be less critical than previously assumed; rather, it is the presence of emojis that drives perceptions of partner responsiveness and subsequent relational outcomes. This challenges the notion that facial or highly expressive emojis are more effective than non-face or less expressive emojis and implies that even simple or abstract emojis can fulfill relational functions in digital communication. The results indicate that the mechanism enhancing relationship perceptions operates independently of emoji type, provided the emoji contributes to an emotionally coherent message.

It is important to note that emojis do not always have a uniformly positive effect. Their interpretation depends on the emotional tone of the accompanying text. To reduce potential misinterpretations, the emoji–text combinations in this study were carefully designed to be emotionally congruent: positive emojis were paired with positive text, negative emojis with negative text, and neutral emojis with neutral text. This congruency aimed to create coherent emotional expressions and minimize ambiguity or sarcasm in interpretation. Therefore, when interpreting the results, it is crucial to consider that these findings reflect the effects of emotionally aligned emoji–text pairings. Different outcomes may arise in situations where emoji and text valence diverge.

The study highlights the significant role of emoji use in shaping individuals’ perceptions of their communication partner’s responsiveness, closeness, and relationship satisfaction. Interestingly, even simple emoji use appears to trigger a positive chain reaction in mutual interactions. Emojis, as ready-made visual cues, enhance communication efficacy in digital interactions by conveying emotional tone, expressiveness, and warmth. By complementing textual communication, emojis improve clarity and emotional depth.

Previous research supports the idea that emojis function as digital equivalents to facial and nonverbal expressions [113], allowing emotions to be conveyed more effectively in text-based communication. Emojis serve as a low-cost and efficient tool for enhancing emotional clarity online. Furthermore, this study found that emoji use significantly improved perceptions of partner responsiveness compared to non-emoji use, regardless of the emoji type—whether facial or non-face.

Several factors may explain why participants perceived partners using emojis as more responsive. First, emojis convey emotions that can be difficult to express through text alone, making messages feel more personal and thoughtful [71]. Second, emojis enhance the emotional appeal of messages, evoking stronger emotional responses [68] and making conversations livelier and more engaging. Third, they help clarify the tone and intent of a message [9,10], reducing the risk of misunderstandings. Lastly, the visual appeal of emojis draws attention to messages, enhancing their expressiveness and enthusiasm.

By exploring how emoji use affects perceptions of a partner’s responsiveness and its subsequent impact on relational outcomes, this study makes a few meaningful contributions to understanding interpersonal processes in digital interaction. First, the findings bridge the gap between text-based communication and face-to-face interactions, suggesting that emojis make digital communication more engaging and effective. Second, regardless of the specific type of emoji, their use consistently enhanced perceptions of responsiveness, likability, closeness, and relationship satisfaction. Finally, the study underscores the centrality of partner responsiveness in shaping interpersonal relationships across both digital and face-to-face contexts.

Emoji uses evoke several practical implications for mutual relationships. First, the findings suggest that incorporating emojis into text messages can signal attentiveness and emotional engagement. For example, adding a heart, smiley face, or other simple emoji can make messages feel more personal and engaging, fostering stronger connections between friends or partners. Second, since the specific type of emoji is less critical than its presence, individuals can benefit from using emojis without overanalyzing their choices. Simple symbols like thumbs-up or sparkles can effectively convey positivity and engagement, even in casual conversations. Third, the mediating role of responsiveness suggests that emoji use enhances the overall quality of digital interactions. This insight is especially valuable in informal contexts, where quick and spontaneous exchanges dominate. Emojis can efficiently convey emotional depth, reducing misunderstandings and enriching the communication experience.

### Limitations and directions for future research

This study has several limitations, suggesting directions for future research. First, this study focused on one partner’s perceptions of relational outcomes, overlooking the inherently dyadic nature of relationships. Although imagined scenarios can elicit meaningful responses, they do not fully capture the emotional depth, interpersonal dynamics, or contextual nuance of real-time exchanges. Additionally, examining only one partner’s perceptions neglects the inherently dyadic nature of relationships. Future research should consider using live asynchronous message-based interactions between individuals to improve ecological validity and provide a more comprehensive understanding of mutual responsiveness and relational dynamics. Prior work has shown that mutual support has been linked to improved mood and reduced negative emotions [54], highlighting the importance of capturing both partners’ perspectives. Examining both partners’ perspectives with live and asynchronous message-based interactions would provide a more complete understanding of how emojis shape relationship processes.

Second, the use of MTurk participants may not fully capture the nuances of communication between close, real-life friends. While MTurk provided a diverse participant pool, future studies could recruit real-life friend pairs to validate and expand upon these findings. Furthermore, participants were also likely more familiar with digital communication, which could influence how they use and interpret emojis. The sample skewed toward middle-aged adults, which may affect generalizability, as younger or older individuals might engage with emojis differently. Additionally, participants were based in the U.S. and completed the study in English. Because emoji meanings can vary by culture, language, and region, these findings may not apply universally. Given the sample’s geographic and linguistic characteristics, findings should be interpreted within the U.S. context, as results may differ across cultures and language backgrounds. Future research should include a broader range of participants, including real-life friend pairs, people with different levels of online experience, and individuals from more diverse cultural backgrounds.

Third, this study adopted a cross-sectional design, which limits the ability to assess the long-term effects of emoji use on relational maintenance. Future research could examine how emoji use evolves over time and its impact across different relationship stages, from casual dating to long-term commitment.

Fourth, another promising direction is the exploration of emoji congruency, i.e., whether emojis align with or contradict the accompanying text (e.g., sarcasm). Incongruent emojis have been shown to reduce message clarity [114]. Future studies could investigate how individual factors, such as emotional intelligence or relationship duration, influence interpretations of emoji congruency.

Finally, while emojis provide accessible and standardized tools for expressing affect and relational intent, their standardized design may introduce limitations. Variations in emoji display across platforms and devices can obscure or distort intended meaning, particularly when users attempt to communicate subtle or culturally specific emotions. Future research should consider how users navigate these limitations and whether they adapt their communication strategies, such as by combining emojis with text, to reduce ambiguity and improve clarity in digital interactions.

### Conclusion

The present study presents the results of an experiment designed to explore the effects of emoji use in text messaging on friendship outcomes. Results revealed that emojis-and-text (compared to text-only) were judged to be more responsive, and that partner responsiveness mediated the effects of emojis on relationship outcomes. These results provide empirical support for the communicative function of emojis in enhancing relational perceptions.

Emojis have become an integral part of today’s digital communications, yet their relational impact remains underexplored in experimental research. This study contributes to the growing body of work on computer-mediated communication by using an experimental design to isolate the effects of emoji use on friendship dynamics – moving beyond the correlational and cross-sectional approaches that dominate much of the existing literature. By highlighting the mediating role of perceived responsiveness, the present study offers a theoretically grounded explanation for why emojis matter in relational contexts: they serve not only as expressive devices but also as cues that signal attentiveness and emotional engagement.

Furthermore, the study underscores the role of emojis as tools that help bridge the gap between the limited emotional bandwidth of text-based communication and the richness of FtF interaction. These findings carry important implications for understanding how digital communicators convey relational intent, particularly in contexts where tone and affect are otherwise difficult to interpret.

By foregrounding partner responsiveness as a key mechanism and demonstrating how even small design features like emoji inclusion can shape interpersonal perceptions, this study lays critical groundwork for future research. It opens avenues for examining how emojis function across various relationship types, cultural contexts, and communication platforms, ultimately enriching our understanding of relational processes in the digital age.
